# Academical Proceedings, National and Foreign

**Published:** 1822-02

**Authors:** 


### ACADEMICAL PROCEEDINGS,

### NATIONAL AND FOREIGN.

### 1. ROYAL SOCIETY OF LONDON.

THE Society met on the 10th of January, after the Christmas adjournment, for the transaction of business.

Dr. WOLLASTON read an extract of a letter from Captain HALL, giving an account of a comet seen at Valparaiso, in April, 1821; and a calculation of the elements of that comet was also presented from Dr. BRINKLY, who thinks it to be the same comet observed in 1793.

At the meeting of the 17th, the same distinguished philosopher read a memoir on the Ultimate Atoms of Matter, tending to show that matter is not infinitely divisible.

A paper was then read, in part, by J. IVORY, Esq. on the Attraction of Spheroids, and the differential Equations that take place at their surfaces.

### 2. ROYAL COLLEGE OF SURGEONS OF LONDON.

*Jacksonian Prize.*—Not either of the prizes for the year 1820 having been adjudicated, *three* prize subjects are proposed for the year 1822, viz.

Injuries and Diseases of Muscle;—Diseases of the Skin;—and the Diseases of the Rectum.

Candidates to be members of the College. Dissertations to be in English; and the number and importance of facts will be considered principal points of excellence. Each dissertation to be distinguished by a motto or device, and accompanied by a paper, sealed up, containing the name and address of the author, and having, on the outside, a motto or device corresponding with that on the dissertation. Dissertations to be addressed to the secretary, and delivered at the College before Christmas-day, 1822.

The prize-dissertations will be preserved in the library of the College. Compositions which shall not be approved, with their correspondent sealed papers, will be returned upon authenticated application; and those which shall remain three years unclaimed, will become the property of the College; at which period their accompanying papers will be burnt, unopened, in the presence of the Jacksonian committee.

### 3. SOCIETY OF MEDICINE OF PARIS.*

This Society proposes, for the subject of a prize-dissertation, founded by Dr. DENEUX, physician-accoucheur to H. R. H. the Duchess de Berry, the following questions:

"To determine what are the complaints which are really the effect of pregnancy;—those which are aggravated by it;—those, the progress of which it suspends; and those that are caused by it."

* This Society must not be confounded with the Royal Academy of Medicine, nor with the Society of the Faculty of Medicine; the two highest medical societies in Paris.

The dissertations must be addressed to Mr. NACQUART, secretary-general to the Society, Rue St. Avoie, No. 30, before the 30th of September, 1822; after which the prize, to consist of a gold medal, value 300 francs, will be adjudicated by the council.

### 4. ATHENEUM OF MEDICINE OF PARIS.

A prize, value 200 francs, will be adjudicated by this Society, in 1822, to the best dissertation on the following question:

"To ascertain, by experiments, as well as by observations, the action of camphor on the human economy, both in a state of health and disease; and to deduce from them the therapeutical properties of this medicine."

The dissertations must be sent to Mr. DE LENS, secretary-general at the Atheneum, Rue Michel-le-Comte, No. 18.

### 5. ROYAL ACADEMY OF SCIENCES OF FRANCE, (INSTITUTE.)

A gold medal, value 3000 francs, will be awarded, at a public meeting of the Academy, in the year 1823, to the author of the best essay on the following subject:

"To determine, by means of accurate experiments, the causes, whether chemical or physiological, of animal heat."

It is expected that the author shall determine, in the most exact manner, the quantity of heat evolved, in a given time, by an animal in health, as well as of the carbonic acid, produced during respiration; and that the quantity of animal heat, thus found, be compared to that produced by the combustion of carbon, for the formation of an analogous quantity of carbonic acid.

The dissertations must be delivered in before the 1st of January, 1823, at the secretary's office at the Royal Institute.

